# Improved Ionic Transport Using a Novel Semiconductor Co_0.6_Mn_0.4_Fe_0.4_Al_1.6_O_4_ and Its Heterostructure with Zinc Oxide for Electrolyte Membrane in LT-CFCs

**DOI:** 10.3390/nano13121887

**Published:** 2023-06-19

**Authors:** Yiwang Dong, Naveed Mushtaq, Muhammad. A. K. Yousaf Shah, Muhammad Yousaf, Yuzheng Lu, Peng Cao, Qing Ma, Changhong Deng

**Affiliations:** 1 School of Electrical Engineering and Automation, Wuhan University, Wuhan 430072, China; 2 Jiangsu Provincial Key Laboratory of Solar Energy Science and Technology/Energy Storage Joint Research Center, School of Energy and Environment, Southeast University, No. 2 Si Pai Lou, Nanjing 210096, China; 3 College of Electronic Engineering, Nanjing Xiaozhuang University, Nanjing 211171, China

**Keywords:** spinel-structured Co_0.6_Mn_0.4_Fe_0.4_Al_1.6_O_4_ (CMFA), semiconductor ZnO, CMFA–ZnO heterostructure composite, higher fuel cell performance, ionic transport, LT-SOFC electrolyte

## Abstract

Improving the ionic conductivity and slow oxygen reduction electro-catalytic activity of reactions occurring at low operating temperature would do wonders for the widespread use of low-operating temperature ceramic fuel cells (LT-CFCs; 450–550 °C). In this work, we present a novel semiconductor heterostructure composite made of a spinel-like structure of Co_0.6_Mn_0.4_Fe_0.4_Al_1.6_O_4_ (CMFA) and ZnO, which functions as an effective electrolyte membrane for solid oxide fuel cells. For enhanced fuel cell performance at sub-optimal temperatures, the CMFA–ZnO heterostructure composite was developed. We have shown that a button-sized SOFC fueled by H_2_ and ambient air can provide 835 mW/cm^2^ of power and 2216 mA/cm^2^ of current at 550 °C, possibly functioning down to 450 °C. In addition, the oxygen vacancy formation energy and activation energy of the CMFA–ZnO heterostructure composite is lower than those of the individual CMFA and ZnO, facilitating ion transit. The improved ionic conduction of the CMFA–ZnO heterostructure composite was investigated using several transmission and spectroscopic measures, including X-ray diffraction, photoelectron, and UV–visible spectroscopy, and density functional theory (DFT) calculations. These findings suggest that the heterostructure approach is practical for LT-SOFCs.

## 1. Introduction

In recent years, the era of energy storage and energy conversion devices, including battery and fuel cells, remained the focus of mitigating the energy crisis and environmental pollution. The main concern is SOFCs (solid oxide fuel cells) converting the chemical energy of storage multi-fuels into electrical energy with zero pollution and remarkably better efficiency. Two main benefits of SOFCs include the usage of multi-fuels and flexibility in modularity for energy conversion technology, which has recently attracted immense attention [1,2,3]. However, SOFCs still encounter bottlenecks of high operational temperature, usage of expensive components, and instability while in operation, which are the main barriers to commercialization. The main issue arises due to ohmic losses of the electrolyte layer as, until now, YSZ is considered the most up-to-date electrolyte with a high ionic conductivity of 0.1 S/cm at 800–1000 °C [4,5]. Additionally, LSGM (La_0.8_Sr_0.2_Ga_0.83_Mg_0.17_O_2.815_) and GDC (Gd_0.1_Ce_0.9_O_2_) have been designed to reduce the operating temperature to 600–1000 °C and maintain high ionic conduction [6,7]. However, until now, these single-phase electrolytes operate at high temperatures which is the main bottleneck to the commercialization of SOFCs. Alternative approaches and technologies, such as thin films, were developed and adopted to reduce the operating temperature and improve ionic conductivity [8,9]. Still, the high cost of manufacturing and scaling up the device hinder the progress of SOFC technology.

Recently, a new semiconductor-based fuel cell technology approach uses electrolytes and electrodes to replace conventional electrolytes and electrodes and mitigate the above-stated concerns. The semiconductor-based fuel cell uses semiconductor ionic materials (SIMs), which possess both ionic and electronic conduction while maintaining better and stable OCV operation with high performance. Most importantly, SIMs can operate at low operational temperatures without power loss and short-circuiting issues [10,11,12]. In this regard, researchers designed a new semiconductor electrolyte for successful operation at low working temperatures. For example, single-phase material semiconductor perovskite SFT (SrFe_0.2_TiO_3_) materials were used as an electrolyte for the application of ceramic fuel cells. It delivered high ionic conduction and an excellent fuel cell performance of 534 mW/cm^2^ at 520 °C. The Schottky junction phenomenon was proposed to suppress the electronic conduction and excel the ions [13,14]. Similarly, Zhuo and Chen have designed new semiconductor electrolytes SmNiO_3_ and SrTiO_3_ and attained maximum fuel cell performance at the lower temperatures of 500 and 550 °C [15,16]. Moreover, many single-layer semiconductor electrolytes have been reported to explore high ion transport with excellent device performance and no short-circuiting issues [17,18,19,20].

Moreover, the semiconductor ionic composite heterostructure approach was adopted at low operational temperatures to boost the ionic conduction and fuel cell performance. Xia et al. used triple conducting semiconductor perovskite BaCo_0.4_Fe_0.4_Zr_0.1_Y_0.1_O_3−δ_ (p-type semiconductor) as an electrolyte via compositing with ZnO (n-type semiconductor) to develop a p-n (BCFZY–ZnO) heterojunction. The constructed heterojunction delivered a peak power density of 643 mW/cm^2^, a better OCV of 1.01 V, and higher ionic conductivity of 0.21 S/cm at 550 °C [21]. Shah et al. designed a composite p-n heterojunction using SFT–ZnO as an electrolyte for the SOFC. The reported electrolyte delivered a high ionic conductivity of 0.21 S/cm and excellent fuel cell performance of 656 mW/cm^2^ with an OCV of 1.06 V at 520 °C [22]. Additionally, a new semiconductor heterojunction was synthesized using SFT (p-type) and WO_3_ (n-type) to shape into an SFT–WO_3_ p-n heterojunction which delivered an excellent power output of 875 mW/cm^2^ and high ionic conductivity of 0.2 S/cm at 520 °C [23]. In addition, other semiconductor materials have a different structure (spinel and perovskite structure) and better electrical properties, especially ionic conduction, even though they are not used in SOFCs as an electrolyte. For instance, Al doping into the B-site of the spinel structure will create excess O-vacancies, enhancing ionic conduction [19,24]. Furthermore, compositing the different structures makes more active sites with enriched O-vacancies, significantly favoring high ionic conduction [21,22,23,25,26]. The above-stated findings evidence that semiconductor technology holds significant potential as a low-temperature SOFC electrolyte regardless of the lower performance concerns.

Based on the above-stated literature on semiconductor technology and the advantages of composite heterostructure semiconductors, we have designed a new homogenous composite semiconductor p-n heterojunction of CMFA–ZnO as an electrolyte where CMFA is a p-type semiconductor while ZnO is an n-type semiconductor. The prepared composite heterostructure was well sandwiched between two symmetrical electrodes of Ni foam NCAL (Ni_0.8_Co_0.15_Al_0.05_LiO_2−δ_) to shape the fuel cell device in the following configuration: Ni-NCAL/CMFA–ZnO/NCAL-Ni. It delivered high ionic conductivity and appreciable power output at a low operating temperature of 450–550 °C. The attained high ionic conduction and better performance realized better functionality via constructing the p-n heterojunction, further suppressing the electronic conduction and promoting the ionic transport. Moreover, our investigation includes preparing and testing the feasibility of CMFA–ZnO electrolyte and performing different characterizations like XRD, SEM, HR-TEM + EDS mapping, UV–visible spectroscopy, and XPS. The attained results and new approach lead to insight from semiconductor and junction perspectives to the design of advanced electrolytes.

## 2. Materials and Methods

### 2.1. Preparation of CMFA–ZnO Heterostructure Composite

#### 2.1.1. Preparation of Co_0.6_Mn_0.4_Fe_0.4_Al_1.6_O_4_

Spinel-structure sol-gel processing using citric acid and ethylenediaminetetraacetic acid (EDTA) as complexing agents yielded CMFA residues. Step one involved dissolving 0.1 moles of EDTA in de-ionized water and adding NH_3_ to increase the pH up to 7.0 and make the solution transparent. After that, the proper proportions of Co (NO_3_)_2_.6H_2_O, Mn (NO_3_)_2_.6H_2_O, Fe (NO_3_)_2_.6H_2_O, and Al (NO_3_)_2_.9H_2_O (99.98%, Alfa Aesar) were added into the homogenous solution. Finally, the solution was stirred at 80 degrees Celsius, 240 revolutions per minute (rpm), for another 6 h, and a brownish gel of CMFA was formed. The resulting brownish gel was then aged for 24 h at room temperature before being dried in an oven set at 150 degrees Celsius. Once dry, the gel was ground into a powder and calcined in the air at 850 degrees Celsius in a muffle furnace for 8 h.

#### 2.1.2. Preparation of Superlattice Heterostructures

The commercially purchased ZnO powder was used as a heterostructure component with CMFA powder with different molar ratios of 9:1, 8:2, 7:3, 6:4, and 5:5. In detail, both powders (ZnO and CMFA) were mixed in a motor pestle and ground well for 2 h. After obtaining both CMFA and ZnO powders, the mixtures were prepared via a solid-state mixing procedure by ball milling the synthesized CMFA and ZnO powders in different weight wt% ratios for 10 h at 400 rpm. Ethanol was used as a dispersing medium. The mixed powders were then sintered at 800 °C for 2 h and ground to obtain CMFA–ZnO.

### 2.2. Experimental Characterizations

A Bruker D8 (X-ray diffractor meter) using Cu-Kα radiation (λ = 1.5418 Å) was used for measuring the XRD patterns of the ZnO, CMFA, and CMFA–ZnO heterostructure composite. The surface morphologies of ZnO, CMFA, and CMFA–ZnO were studied using a scanning electron microscopy of Merlin compacts (Zeiss). The Raman analyses of CMFA, ZnO, and CMFA–ZnO were performed using an NT-MDT (Russia) spectrometer with a 532 nm solid laser source. The surface properties of CMFA, ZnO, and CMFA–ZnO were studied using an X-ray photoelectron spectroscopy (Physical Electronics Quantum 2000) with an Al Kα source Gamry Reference 3000 (pine instruments the U.S), and ZSIMPWIN software was used to measure and analyze the electrochemical impedance data in the open-circuit voltage condition in the 0.1 HZ to 1 MHz frequency range. A dc signal of 10 mV was applied to record the data.

### 2.3. Device Fabrications and Electrochemical Measurements

The practical applicability of CMFA and its heterostructure composite with ZnO as an electrolyte in a ceramic fuel cell and the fabrication of CFC devices were arranged by dry pressing. As-prepared CMFA, ZnO, and CMFA–ZnO heterostructure composite electrolyte membranes were pressed between two symmetrical Ni_0.8_Co_0.15_Al_0.05_LiO_2−δ_ (NCAL from Bamo Sci. & Tech. Joint Stock Ltd.) electrodes. First, a slurry of the NCAL was made in terpinol and was brushed onto Ni-foam, followed by a desiccation process at 120 °C for 1 h. Afterwards, a dried piece of NCAL-painted Ni-foam was added in a steel dye, followed by CMFA, ZnO, or CMFA–ZnO electrolyte powders (0.20 g) and another piece of NCAL-painted Ni-foam. However, the three-layered structure of the cell was pressed unanimously at 200 MPa. The same protocol was applied to prepare all cells. Thus, in the end, we got different ceramic cells to measure the performance, such as Ni-foam NCAL/CMFA/Ni-NCAL, Ni-foam NCAL/ZnO/Ni-NCAL, and Ni-foam NCAL/CMFA–ZnO/Ni-NCAL for comparative study. The performance demonstration of a single CFC was done using 3% H_2_O-humidified hydrogen. The H_2_/air pressure was set to 100–120 ± 5% mL/min.

### 2.4. Computational Methods

Spin-polarized DFT calculations on individual CMFA and CMFA–ZnO heterostructure composite were carried out using CASTEP (Cambridge serial total energy package) on Materials Studio 8.0. within the projector-augmented wave (PAW). The cubic spinel structure of CMFA (a = b = c = 0.06551 nm and α = β = γ = 60°) with space group Fm-3m-22-1086 and hexagonal structure ZnO with space group Fm-3m-225 and lattice parameters a = b = c = 5.4130 Å were used to optimize the crystal structures and their properties. The generalized gradient approximation and Perdew–Burke–Ernzerh (GGA + PBE) method were used to control the electron exchange correlation energy. Meanwhile, the LDA + U method was used with U = 4.5 eV for Fe-3d and U = 5.6 eV for Ce-4f. The energy cutoff for the plane-wave basis set was 720 eV, and the PAW data sets were used with the following valence electronic states: Fe (4s2, 4p6 5s2), and O (1s2, 2s2, 2p4). The slab model was applied to construct the CMFA–ZnO heterostructure, and each slab of the CMFA surface and ZnO layer were separately computed in advance. A √2 × 3√2 × 1 supercell structure was created comprising (3 × 3 × 2) surface units for ZnO (111) and (2 × 2 × 2) surface units of CMFA (001) with minimized lattice mismatch <5%. A sufficient vacuum was applied to protect the self-interaction in the z-direction. The oxygen vacancy formation energy on CMFA, ZnO and CMFA–ZnO surface was calculated using [27,28]:

EV_o_ = E_tot_ (V_o_^q^) − E_tot_ (ideal) + μ + q(E_F_ + E_valan_ + ∆V)

where EV_o_ is the vacancy formation energy, E_tot_ (V_o_^q^) is the superlattice total energy in q charge state, and E_tot_ (ideal) is the total energy of the ideal structure. Additionally, μ is the chemical potential and E_F_ is the orientation of the Fermi level. E_valan_ refers to the valence band maximum and ∆V represents changes in the electrostatic potential with defective lattice.

## 3. Results and Discussion

### Structural and Cation Arrangement

**Figure 1:**
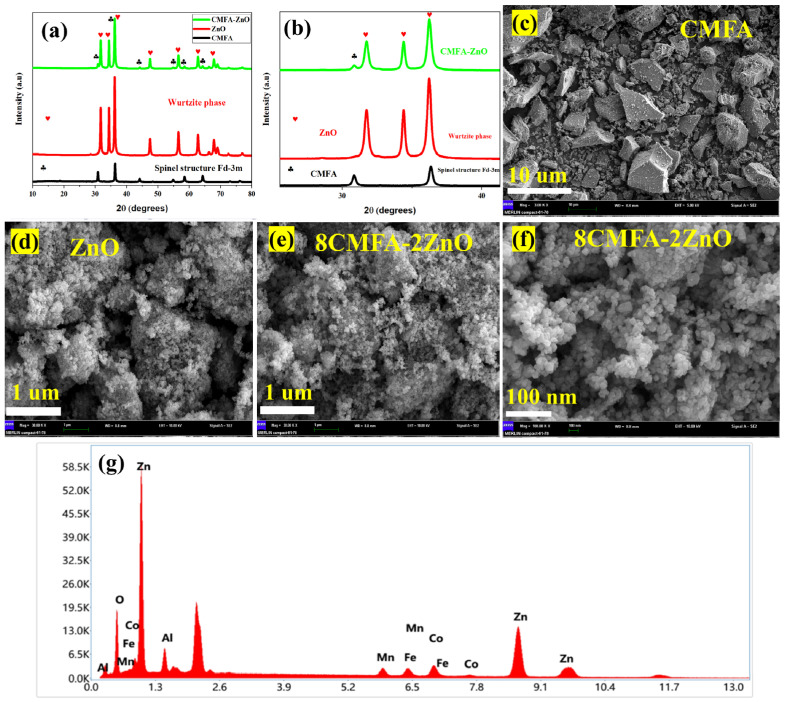
(**a**) XRD pattern of CMFA as synthesized by the sol-gel method, the XRD pattern of commercially purchased ZnO, and the XRD pattern of heterostructure CMFA–ZnO as synthesized by ball-milling technique, (**b**) along with a magnified view of pure CMFA, pure ZnO, and CMFA–ZnO heterostructure samples. (**c**–**f**) SEM images of the CMFA, ZnO, and 8CMFA–2ZnO heterostructure composite, respectively. (**g**) EDS energy spectrum of 8CMFA–2ZnO.

Figure 1a shows the XRD pattern of the prepared spinel-structured CMFA, ZnO, and CMFA–ZnO heterostructure composite in 2θ of 10–80°. The dominant diffraction peaks of CMFA are located at 2θ of 18°, 30°, 34°, 36°, 42°, 52°, 56°, 62°, 65°, and 78°, which can be directed to the (111), (220), (311), (222), (400), (422), (511), (440), (620) and (533) planes, respectively (Figure 1a), corresponding to Fm-3m space group (JCPDF # 22-1086) [29,30]. The measured XRD pattern is a pure cubic spinel structure. The prominent diffraction peaks of ZnO are located at 31.7°, 34.4°, 36.2°, 47.4°, 56.4°, 62.8°, and 67.7°, belong to the indices of (100), (002), (101), (102), (110), (103), and (112), respectively, which are well-matched with the previously published reports [22]. The XRD pattern of ZnO confirmed the formation of a pure hexagonal structure which agrees with the published reports. The diffraction pattern of the CMFA–ZnO heterostructure composite showed a mixed phase of hexagonal and fluorite structure (ZnO and CMFA), indicating the coexistence of both diffraction patterns. No peaks other than CMFA and ZnO diffraction patterns were observed in the CMFA–ZnO heterostructure composite sample, eliminating the possibility of any chemical reactions forming new phases. Figure 1b reveals the magnified view of the XRD pattern of CMFA, ZnO, and CMFA–ZnO, confirming the formation of a pure structure without any impurities and chemical reactions.

Furthermore, Figure 1c–f shows the SEM (scanning electron microscopy) of the CMFA, ZnO, and CMFA–ZnO heterostructure composite. The SEM morphology of CMFA appeared to be rock-type, where particles were asymmetrically distributed at the micro-scale, as depicted in Figure 1c. Figure 1d displays the SEM image of ZnO where particles were in irregular shape where particles were randomly distributed with smaller and larger particles. The SEM morphology of composite heterostructure CMFA–ZnO is shown in Figure 1e,f, where grain levels interaction of each composition can be seen with sophisticated and fine particles with nano-sized particles, indicating the growth of particles in heterostructures as previously reported [31]. The strong interconnections between CMFA–ZnO particles make the high oxygen penetration rate possible. Because of this characteristic, the prepared composite meets the necessary criteria for electrolyte membranes by creating more active spots for electrochemical reactions and serial interfaces and pathways for charge transport [22]. Moreover, to confirm the elemental distribution at the particle level, the energy dispersive (EDS) sum spectrum was measured using an SEM image as depicted in Figure 1g, where all particles seem to be present in the prepared composite heterostructure CMFA–ZnO.

**Figure 2:**
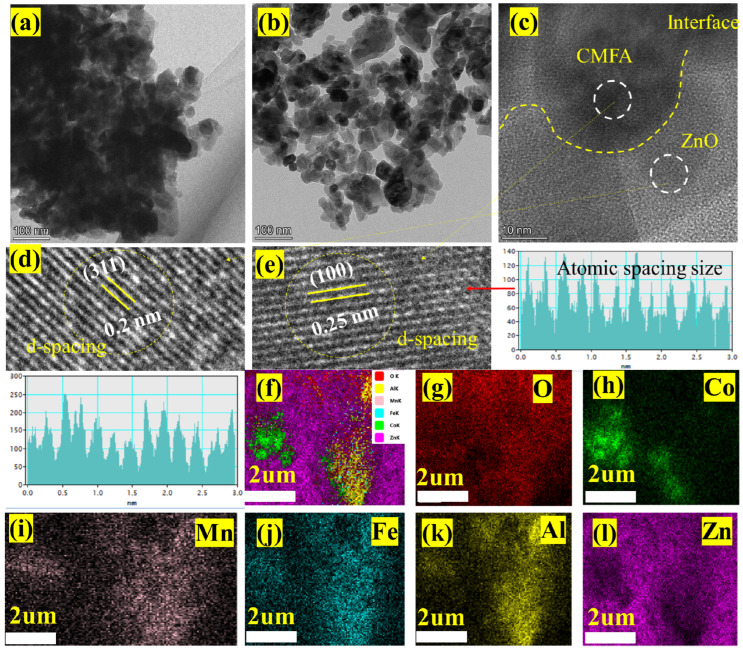
(**a**–**e**) HR-TEM image of composite heterostructure 8CMFA-2ZnO, (**c**) the dotted line indicate the interface between the CMFA and ZnO and (**d**,**e**) d-spacing with different planes of CMFA and ZnO; (**f**–**l**) SEM EDS elemental mapping of composite heterostructure of 8CMFA–2ZnO including all elements (Co, Mn, Fe, Al, Zn and O).

Moreover, the crystallographic structure of the bulk heterostructure composite CMFA–ZnO was determined by HR-TEM, as presented in Figure 2a–e. Figure 2a,b shows the HR-TEM morphology images of the composite heterostructure at the nanoscale of 100 and 500 nm where particles were uniformly dispersed and well-adhered to established a continuous network which assists in overall quick and easy charge transportation leading to enhancement in the fuel cell performance. Figure 2c shows the HR-TEM image of the heterostructure where the particles of CMFA and ZnO seemed too well-connected, and the connection was separated using the interface, confirming the establishment of heterojunctions at particle scale, which is beneficial for fuel cell device performance [26]. Additionally, the grain level interactions of each composition was seen with fine particles. Furthermore, the HR-TEM image of Figure 2c has been modified to attain the d-spacing images of the composite heterostructure where the d-spacing of 0.2 nm is linked to the 311 planes of CMFA while the d-spacing of 0.25 nm connected to the 100 planes of ZnO as depicted in Figure 2d,e. Furthermore, Figure 2f shows the SEM-EDS mapping images where all elements are identified using different colors, confirming all elements’ presence in the constructed composite heterostructure. Additionally, in the composite heterostructure, homogenous chemical spreading of each element, such as Co, Mn, Fe, Al, Zn, and O, can be seen, confirming the formation of a heterostructure composite at particle/grain levels as depicted in Figure 2g–l.

**Figure 3:**
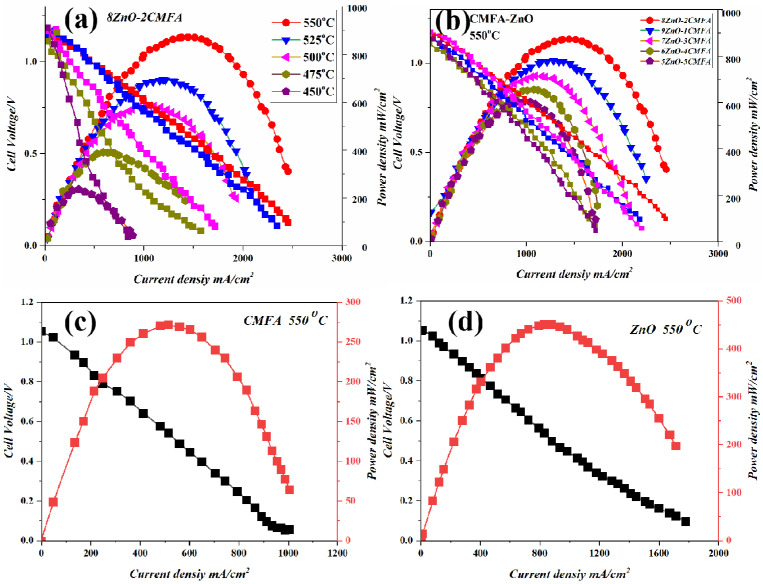
(**a**) I-V and I-P characteristic curve using the best performing 8ZnO–2CMFA electrolyte membrane at different operating temperatures from 450 to 550 °C; (**b**) I-V and I-P characteristics of different CMFA–ZnO heterostructure composite electrolyte membranes at 550 °C; (**c**,**d**) fuel cell performance of CMFA and ZnO at 550 °C.

The SOFCs used single CMFA, ZnO, and CMFA–ZnO heterostructure composites as their electrolytes, and the performance of each was assessed between 450 and 550 °C. The acquired current–voltage (I–V) characteristics for the CMFA and ZnO electrolyte fuel cells, separately at 550 °C, are shown in Figure 3a,b, together with the related current–power (I-P) plots. As shown in Figure 3d, a composite heterostructure using CMFA–ZnO as an electrolyte was used at various operating temperatures ranging from 450 to 550 °C. It has been demonstrated that all materials, including CMFA, ZnO, and CMFA–ZnO heterostructure, can achieve superior electrolyte functioning, showing a favorable current density and having a significant power density.

At 550 °C, the maximum power density of the CMFA and ZnO electrolyte fuel cells was 272 mW/cm^2^ and 450 mW/cm^2^, respectively, with an open circuit voltage (OCV) of 1.02 V and 1.05 V. This was better than what has been reported for semiconductor electrolytes SNO and LCAO [15,32]. A Schottky barrier in the Schottky Junction between the metallic anode Ni and the semiconductors CMFA and ZnO can explain this high OCV. This barrier stops electrons from passing through the anode/electrolyte interface [33]. Additionally, adding low and medium concentrations of Fe to CMA can make CMFA have much better ionic conductivity, which can help ions move quickly through the CMFA electrolyte layer [34]. Moreover, the way ions move through ZnO can be changed by doping it or combining it with another semiconductor and ionic conductor [21,23,26,35].

More appealingly, the CMFA–ZnO electrolyte fuel cell performed much better than the CMFA fuel cell. It had a peak power density of 872, 692, 582, 389, and 235 mW/cm^2^ and a good OCV at different operating temperatures of 550–450 °C. Additionally, these results show that the devices can work at low temperatures with power outputs of 235 mW/cm^2^ at 450 °C, as shown in Figure 3c,d which shows the typical performance of a fuel cell with different compositions (8:2, 9:1, 7:3, 6:4, 5:5 ZnO-CMFA) under the same conditions. At 550 °C, the performance was excellent. The performance of the most appropriate composition 8ZnO:2CMFA was better than the other mixtures, which suggests that CMFA–ZnO could be a suitable electrolyte.

**Figure 4:**
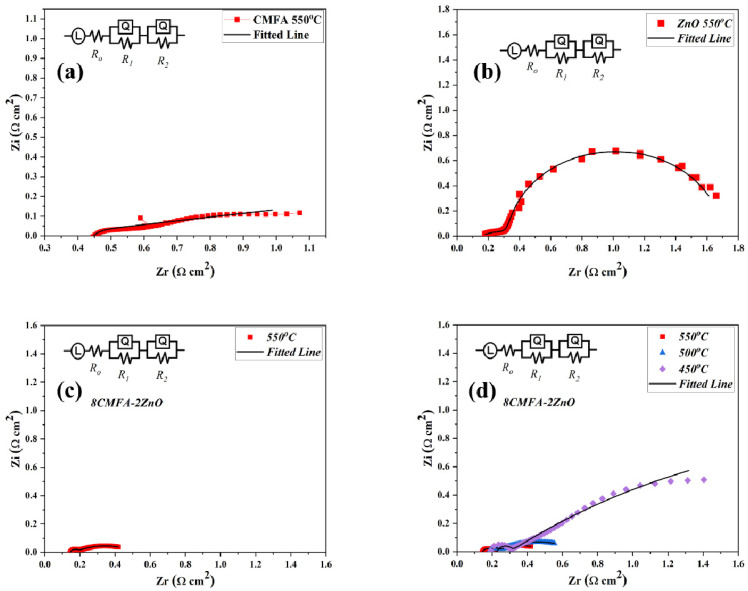
(**a**–**c**) EIS spectra of CMFA, ZnO, and 8CMFA–2ZnO under H_2_/air environment at 550 °C; (**d**) EIS spectra of composite heterostructure 8CMFA–2ZnO under H_2_/air environment at different operating temperatures from 550 to 450 °C.

To determine the electrochemical and electrical properties of CMFA, ZnO, and CMFA–ZnO heterostructure, impedance spectra of CMFA, ZnO, and CMFA–ZnO electrolyte-based fuel cells were measured in an H_2_/air atmosphere at 550 °C and 550–450 °C, as shown in Figure 4a–d. Figure 4 shows the Nyquist plots of the measured EIS data, which were then fit to an equivalent circuit (LR_0_-R_1_Q_1_-R_2_Q_2_) where, R_0_ is the ohmic resistance, R_1_ is the charge transfer resistance, while R_2_ is the mass transfer resistance and Q is the constant phase elements [21]. The EIS curves that were measured show three main processes. The intersections at high frequencies (HF), intermediate frequencies (IF), and low frequencies (LF) show ohmic resistance (R_0_), charge transfer resistance (R_1_), and mass transfer resistance (R_2_). At 550–450 °C, the CMFA–ZnO cell had small ohmic resistances, but at lower temperatures, the polarization resistance was much lower. This means that the charge transfer and mass transfer happened quickly [17,36,37,38]. Additionally, the EIS curves for CMFA and ZnO at 550 °C showed a lower resistance value than for the composite heterostructure (Figure 4a–c). CMFA–ZnO’s total electrical conductivity is made up of both ionic and electronic contributions, as shown by the fitted R_0_ using EIS spectra. Additionally, this low ohmic and polarization resistance was caused by the formation of heterojunctions, a space charge region, and a built-in electric field, all of which helped charge move through the grain boundary interface.

**Figure 5:**
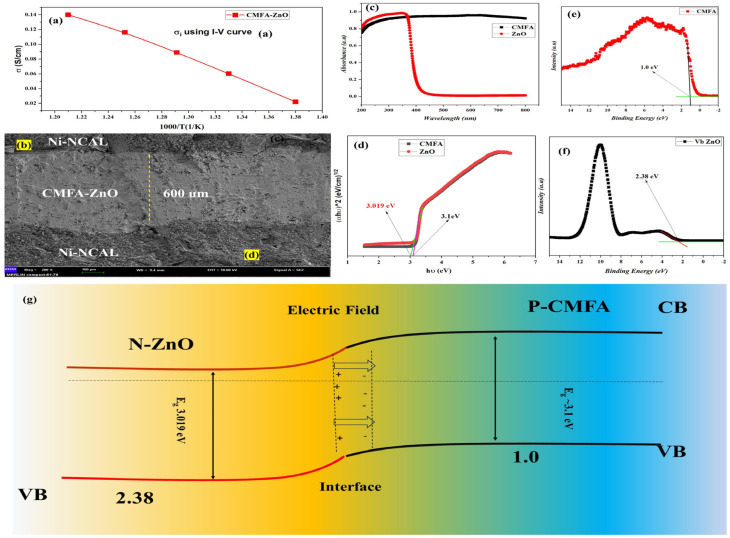
(**a**) Ionic conductivity of 8CMFA−2ZnO at different operational temperatures 550–450 °C, (**b**) SEM cross-sectional view of the 8CMFA−2ZnO pellet after testing, (**c**,**d**) UV–visible spectra of CMFA and ZnO, (**e**,**f**) Vb of CMFA and ZnO while (**g**) is the energy band alignment of composite heterostructure 8CMFA−2ZnO.

**Figure 6:**
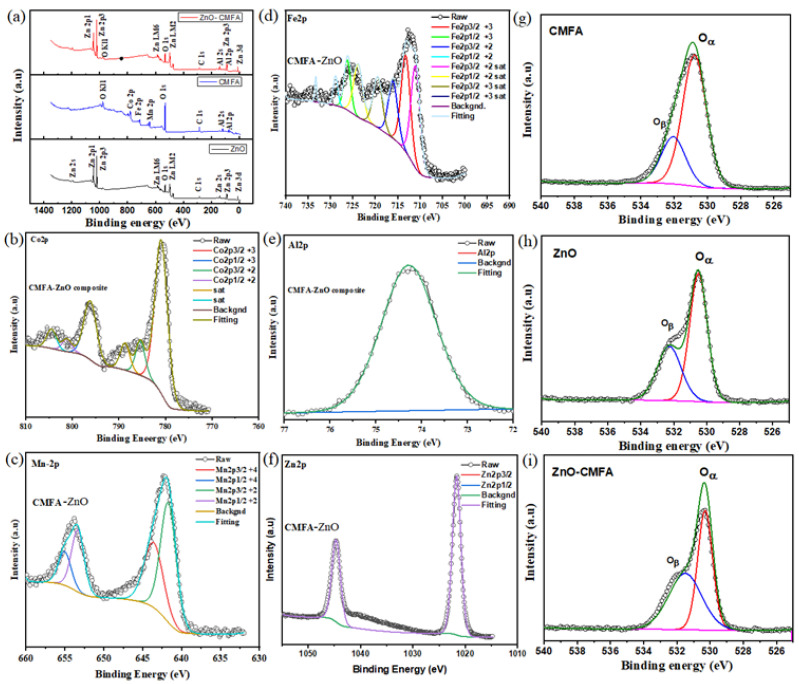
(**a**) XPS survey spectra of CMFA, ZnO and CMFA–ZnO, (**b**–**f**) the XPS spectra of Co-2p, Mn-2p, Fe-2p, Al-2p, Zn-2p, respectively; (**g**–**i**) the O-1s spectra of CMFA, ZnO, and CMFA–ZnO, respectively.

We also believe that the ionic conductivity of the proposed electrolyte CMFA–ZnO at 550–450 °C is essential to the current work. Hence, we employed the unusual approach to calculate this property. The ionic conductivity is evaluated and computed from the I–V curve in Figure 5a, which depicts the ohmic resistance of the polarization curve [18,24,39,40]. The polarization curve’s temperature-dependent linear region results from the electrolyte’s ohmic resistance and the electrodes. When compared to the proposed electrolyte CMFA–ZnO, the resistance provided by the electrode Ni-NCAL is negligible. The ionic resistance of an electrolyte is assumed to be equal to the resistance obtained using the RASR = Vohm/Iohm (ohmic polarization loss and current drop) formula. After that, we determined the ionic conductivity by plugging the values into the formula = L/RA, where L is the electrolyte layer thickness, R is the ohmic resistance, and A is the cross-sectional area. Using a different temperature range of 550–450 °C, the ionic conductivity was predicted to be 0.14–0.08 S/cm (Figure 6a). The achieved ionic conductivity was more significant than that reported in the literature for single-phase and composite-based materials such as GDC (0.0058 S/cm at 500 °C), YSZ (0.0011 S/cm at 500 °C), SFT–SDC (0.1 S cm^−1^ at 520 °C), and SiC–ZnO (silicon–carbide—zinc oxide; 0.12 S cm^−1^ at 550 °C) [4,7,41,42]. The superior ionic conductivity can be primarily attributed to the well-engineered composite heterojunction used in the experiment.

Furthermore, the absorbance spectra were measured using the UV–visible absorbance spectra, as depicted in Figure 5b. The direct bandgap was measured using the Tauc-plot about 3.1 and 3.01 eV that corresponds to the CMFA and ZnO, respectively, as illustrated in Figure 5e [22]. Moreover, the XPS + Vb was used to determine the valence position as about 1 eV and 2.38 eV that corresponds to the CMFA and ZnO, respectively, as illustrated in Figure 5c,f. Figure 5d depicts the SEM image of the CMFA–ZnO fuel cell device following the procedure. It reveals the CMFA–ZnO layer sandwiched between the two NCAL-Ni electrodes. Figure 5d illustrates the anode layer’s porous structure and the CMFA–ZnO layer’s dense structure, which assures an efficient electrode reaction and sufficient OCVs.

The conduction band (CB) levels were determined using the formula Eg = Vb + Vc in conjunction with bandgap and valence band measurements. The CMFA and ZnO have distinct energy band level parameters; when CMFA and ZnO particles come into contact to form hetero-phasic interfaces, their distinct Fermi energy levels will converge to a continuous state at the interface region. Thus, the CMFA and ZnO can produce a p-n bulk heterojunction at the particle level in the composite. To depict the junction effect on electrochemical enhancement (ionic promotion) and charge separation (electron-blocking) process, Figure 5g illustrates the energy band alignment of the CMFA–ZnO p-n junction. Positive and negative charges typically redistribute at the interface of p/n semiconductors to provide an inherent field in a p-n junction (space charge region) [43,44]. In the case of CMFA–ZnO p-n bulk heterojunction, the electric field was able to prevent electrons from passing through, resulting in suppressed electronic conduction throughout the CMFA/ZnO interface; on the other hand, oxygen ions could be motivated and accelerated by the electrostatic force to gain enhanced conductivity, improved the OCV, and decreased activation energy [7,38]. Based on this mechanism, it is reasonable to understand the greater power density of the CMFA–ZnO cell compared to the CMFA and ZnO cells: the ionic enhancement process primarily occurred at the grain boundary interface between CMFA and ZnO. Therefore, the grain-boundary ionic resistance would be decreased, resulting in a high grain-boundary ionic conductivity of CMFA–ZnO. In addition, the electrical blocking behavior was limited to the heterostructure electrolyte layer. It did not occur at the electrolyte/electrode interface, indicating that sufficient electrons can participate in the electrode reaction, resulting in a low polarization resistance [10].

Furthermore, as shown in Figure 6, XPS analysis was used to investigate the chemical states and surface chemical composition of the proposed materials CMFA, ZnO, and CMFA–ZnO. All elements, including Co, Mn, Fe, Al, Zn, and O, were detected in the XPS spectra of CMFA, ZnO, and CMFA–ZnO, as shown in Figure 6a.

Furthermore, as shown in Figure 6, XPS analysis was used to investigate the chemical states and surface chemical composition of the proposed materials CMFA, ZnO, and CMFA–ZnO. All elements, including Co, Mn, Fe, Al, Zn, and O, were detected in the XPS spectrum investigation of CMFA, ZnO, and CMFA–ZnO, as shown in Figure 6a. Additionally, Co-2p peaks were detected at specific binding energy sites, such as 777 and 798 eV, which are attributed to the 2p3/2 and 2p1/2. Additionally, as shown in Figure 6, two detected peaks were further deconvoluted into four peaks at varied binding energies, including 776 and 780 eV, 796 eV, and 798 eV, which are attributed to the different oxidation states +2 and +3 of cobalt [45]. With a spin-orbit level energy spacing of 11.5 eV and two peaks at 642.2 eV for Mn 2p3/2 and 653.7 eV for Mn 2p1/2, the deconvoluted Mn 2p is typical of Mn^3+^-based materials as shown in Figure 6c [46]. Fe^2+^ 2p1/2 and Fe^2+^ 2p3/2 are at 709.2 and 722 eV, respectively. In contrast, Fe^3+^ 2p1/2 and Fe^3+^ 2p3/2 are located at 710.963 and 725.1 eV, respectively, in the Fe 2p spectra shown in Figure 6d [39]. The Fe^3+^ 2p1/2 and Fe^3+^ 2p3/2 peaks, as well as a satellite peak at 717.9 eV, are likewise connected to the BEs of 712.96 and 725.31 eV, respectively [13,27,47,48]. According to these results, Fe 2p can be found at each BE in one of three possible oxidation states (Fe^2+^ and Fe^3+^), as shown in Figure 6d. The XPS spectra of the composite heterostructure CMFA–Al-2p ZnO’s and Zn-2p (2p3/2 and Zn2p1/2) are also shown in Figure 6e.

As shown in Figure 6, the O-1s spectra of CMFA, ZnO, and CMFA–ZnO have also been examined at various binding energies with three unique peaks (Figure 6f,g). The lattice oxygen corresponds to or is associated with the peak O at 529–530 eV. While the peak, designated as O, is pertinent to the O-vacancies and is centered at approximately 532 eV. O-species adsorbed on O-vacancies or surface O-defects may be responsible for the peak at 532 eV, reflecting oxygen that is not tightly bound [8,9,10,11,12]. The formation of the host-oxygen link during heterostructure building makes the oxygen more likely to aid the process of ions moving from the bulk lattice to the surface and interface lattice, hence boosting surface and interfacial conduction [8,49,50]. Additionally, the development of the heterostructure CMFA–ZnO causes the peak area to be larger than that of CMFA alone and ZnO alone, allowing for the accommodation of more oxygen species and oxygen vacancies and thereby improving ionic conduction [12].

**Figure 7:**
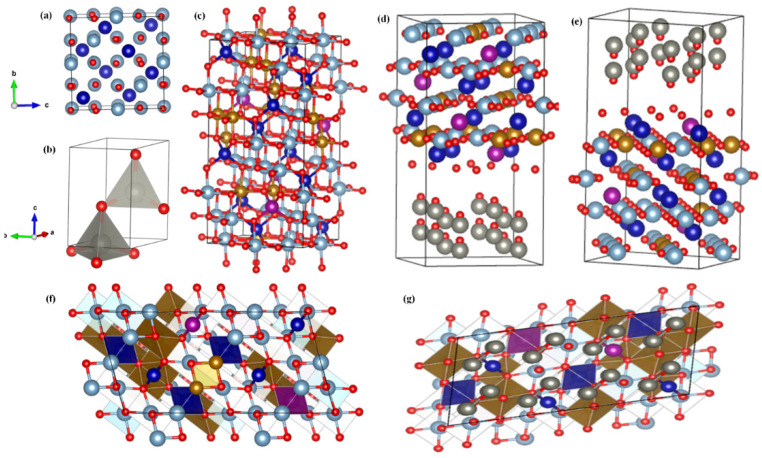
The optimized structure of (**a**) CMFA (110), (**b**) ZnO (110), and (**c**) CMFA–ZnO heterostructure composite (top view CMFA). (**d**) Top view of ZnO in CMFA–ZnO heterostructure composite slab and (**e**–**g**) side standard view of CMFA–ZnO heterostructure composite.

The DFT technique was used to conduct theoretical studies of CMFA, ZnO, and the CMFA–ZnO heterostructure composite to better understand their electrical characteristics and ion diffusion [28]. Figure 7 shows the modelled structures of CMFA, ZnO, and CMFA–ZnO heterostructure composite. As a first step, we independently estimated the structures and characteristics of CMFA (Figure 7a) and ZnO (Figure 7b) to evaluate and contrast them. Our CMFA–ZnO heterostructure composite was thus fabricated using two such refined designs. Figure 7c–g provides a top-down and bottom-up perspective of the possible orientations with CMFA–ZnO heterostructure composite slabs.

**Figure 8:**
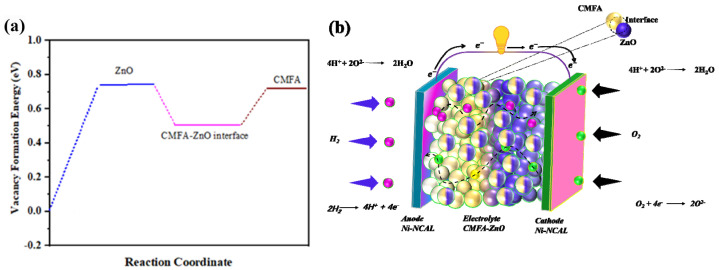
The vacancy formation energy of (**a**) bulk CMFA, ZnO at the interface of CMFA–ZnO and (**b**) schematic diagram of fuel cell based on CMFA–ZnO heterostructure composite electrolyte membrane and mechanism of the ionic transport.

In addition, the electrical properties of a material can be revealed by measuring its energy for oxygen vacancy formation (EVo). Vacancy creation at various sites was used to determine the (EVo) for the CMFA, ZnO, and CMFA–ZnO heterostructure composite (additional information may be found in the experimental section). ZnO and CMFA lattice sites hosted the (EVo) for a different position, with Zn Vo, Co Vo-Fe occurring in the CMFA–ZnO lattice and at the interface. At the CMFA–ZnO heterostructure contacts, the (EVo) was only 0.45 eV, as depicted in Figure 8a. However, the crucial component to disrupt the structure and thus lead to very low EVo energy also disturbed the lattice strain and multivalent oxidation states at the interface of the CMFA–ZnO heterostructure composite. In addition, Figure 8b shows the schematic diagram of a fuel cell device where CMFA–ZnO was sandwiched between symmetrical electrodes of Ni-NCAL with the resumption of ions.

## 4. Conclusions

Enhancing ionic conduction at low temperatures in spinel and hexagonal structure-based ZnO–CMFA heterostructure composites was the focus of this work. Higher ionic and electrochemical performance (872 mW/cm^2^ at 550 °C) was seen in the constructed CMFA–ZnO electrolyte. Many variables contributed to the well-established interface between ZnO and CMFA, responsible for the superior fuel cell performance and high ionic conductivity of the CMFA–ZnO heterostructure composite. Combining theoretical and experimental methods, we examined the mechanism for the transport of high ions. Oxygen vacancies were discovered to be abundant at the CMFA/ZnO interface, allowing for easy ion migration that may increase the self-consistency of the CMFA–ZnO structure and manage the interface-level charge transit capacities for better ionic conduction. The findings show that this strategy can be used to create novel alternatives to existing materials for advanced ceramic fuel cells, where it can improve performance and ionic conduction while also introducing novel functionalities (energy band alignment) and where it may find use in other relevant energy applications, such as photocatalytic water splitting.

## Data Availability

The data supporting this study’s findings are available from the corresponding authors upon reasonable request.
